# Restorative Strategies in Movement Disorders: the Contribution of Imaging

**DOI:** 10.1007/s11910-017-0807-1

**Published:** 2017-11-02

**Authors:** Nicholas P. Lao-Kaim, Paola Piccini, Yen F. Tai

**Affiliations:** Division of Brain Sciences, Charing Cross Hospital, Imperial College London, Fulham Palace Road, London, W6 8RF UK

**Keywords:** Neural transplantation, Emission tomography, Parkinson’s, Huntington’s

## Abstract

**Purpose of Review:**

The purpose of this review was to review the imaging, particularly positron emission tomography (PET), findings in neurorestoration studies in movement disorders, with specific focus on neural transplantation in Parkinson’s disease (PD) and Huntington’s disease (HD).

**Recent Findings:**

PET findings in PD transplantation studies have shown that graft survival as reflected by increases in dopaminergic PET markers does not necessarily correlate with clinical improvement. PD patients with more denervated ventral striatum and more imbalanced serotonin-to-dopamine ratio in the grafted neurons tended to have worse outcome. In HD transplantation studies, variable graft survival and clinical responses may be related to host inflammatory/immune responses to the grafts.

**Summary:**

Information gleaned from imaging findings in previous neural transplantation studies has been used to refine study protocol and patient selection in future trials. This includes identifying suitable candidates for transplantation using imaging markers, employing multiple and/or novel PET tracers to better assess graft functions and inflammatory responses to grafts.

## Introduction

In the last few decades, a tremendous amount of effort has been invested in finding a disease-modifying treatment for various neurodegenerative disorders. Neurorestoration aims to replace the lost or dysfunctional neurons and/or glial cells in the diseased brain. This may be achieved through several means, including (i) administration of neurotrophic factors to promote neurogenesis or (ii) cell replacement therapy/neural transplantation with neuroprogenitor cells [1].

Neural transplantation has been trialled in a number of movement disorders where a predominant cell-type is preferentially lost, such as substantia nigra dopaminergic neurons in Parkinson’s disease (PD) and striatal medium-spiny projection neurons in Huntington’s disease (HD), in an attempt to replace the affected neurons. The outcomes of these studies are somewhat variable. Many human studies have not been able to consistently reproduce the successes seen in initial pilot studies or animal studies.

Functional imaging, particularly positron emission tomography (PET), gives us a unique opportunity to study in vivo the survival and functional integration of implanted cellular grafts. In this review, we will discuss how PET findings in neurorestorative studies help us understand the outcome of completed clinical trials, and how it can help refine and improve restorative strategies for future studies. The review will focus on neural transplantation studies in PD and HD, with some discussions on restorative studies using neurotrophic factors.

### Neural Transplantation in Parkinson’s Disease

Research on intrastriatal transplantation of dopamine-rich human foetal ventral mesencephalic tissue for PD patients began in the late 1980s [2–7] with these and ensuing open-label clinical trials reporting significant symptomatic improvement and reduction in levodopa requirements in several cases [8–34].

PET, utilised mainly in conjunction with dopaminergic radioligand markers, has been a vital tool for objectively assessing post-operative grafted cell function in vivo. In particular, ^18^F-DOPA, a fluorinated analogue of the dopamine precursor l-DOPA which follows the same synthetic pathway of decarboxylation by aromatic amino acid decarboxylase and storage in presynaptic vesicles as ^18^F-labelled dopamine [35, 36], has enjoyed frequent usage for its ability to provide measurements of dopamine synthesis and storage alterations in the grafted striatum. A multitude of early open-label trials have reported elevations of ^18^F-DOPA uptake in transplanted nuclei to within “normal range” as early as 8 months post-transplant, showing averaged increases of 40–120% at 12 months as compared to pre-operative baseline levels. In some cases, the improvements in PET uptake cases were sustained for up to 16 years with associated clinical benefit [8, 11, 13, 15, 16, 19, 25, 30–34, 37–39], demonstrating long-term survival and viability in the host environment. In accordance with this, acute administration of methamphetamine or l-DOPA in transplanted patients has been shown to cause significantly greater reductions in ^11^C-raclopride binding as compared to non-transplanted individuals [29, 37–41]. ^11^C-raclopride is a weak reversible D_2_ receptor antagonist which is displaced when in competition with extracellular dopamine (DA); hence, drug-induced changes with respect to saline can be interpreted as a surrogate marker of DA release. Normalised ^11^C-raclopride binding reductions have been observed from the 5th to the 16th post-operative year [37–39], where even in the worst cases, the percentage decreases were still significantly greater than in non-transplanted putamina [29, 41]. Moreover, changes in ^11^C-raclopride binding were negatively related to changes in ^18^F-DOPA uptake [41]. Collectively, these findings were taken to reflect a functional capability for grafted neurons to release endogenous dopamine and to at least partially restore post-synaptic D_2_ receptor occupancy.

The degree to which the grafted neurons express the dopamine transporter (DAT), a transmembrane protein that facilitates reuptake of extracellular DA, has however been somewhat less consistent across studies. Despite a significant ^18^F-DOPA increase, Cochen et al. found in a group of six patients no change in striatal DAT binding using ^76^Br-FE-CBT PET [42]. In contrast, others have demonstrated large increases in DAT density detectable by 5–6 months after transplant, based on ^11^C-nomifensine binding asymmetry between grafted and non-grafted striata [14] and pre-post-operative changes in ^123^I-IPT binding [43]. Continual rises in ^123^I-IPT and ^123^I-FP-CIT binding have been observed in the long term but not necessarily uniformly across target regions, with one patient sustaining normal levels 14 years after transplant in the clinically least affected striatum but reversal to baseline levels following an initial increase over the first 3 years in the more affected striatum [37, 43], possibly reflecting variability in tissue quality or an effect of lateralised disease severity [43]. Interestingly, changes in ^76^Br-FE-CBT binding and ^18^F-DOPA uptake between scanning sessions were not correlated [42], suggesting that the ability for grafted neurons to synthesise DA is not necessarily related with their capability for re-innervating the host striatum. Despite this, graft-related increases in DAT were accompanied by improvements in general motor severity and vice versa across time points in several studies [14, 42, 43], suggesting that DA terminal integrity is an important factor which may contribute to clinical outcome.

Studies employing non-dopaminergic imaging techniques have provided further supplementary information on graft survival and function. Using proton magnetic resonance spectroscopy, Ross et al. demonstrated that the concentration of *N*-acetylaspartate around the putaminal graft sites, a metabolite marker of neuronal viability and maturity which is not present in foetal neurons, was only marginally shy of pre-operative levels approximately 1 year after surgery, with the lowest detected concentrations still significantly higher than in foetal tissue assayed in vitro [44]. Functional magnetic resonance imaging has revealed arm movement-related activation within these graft sites 2–3 years after surgery [45], indicating sustained functional integration of the foreign tissue following a plateau in graft-related increases of dopamine synthesis. Indeed, while increases in ^18^F-DOPA uptake predominantly occurred within the first 18 months with negligible change beyond this point [8, 11, 22, 46], H_2_^15^O PET demonstrated that the restoration of downstream cortical motor is relatively delayed, illustrating significantly increased cerebral blood flow during left-handed motor joystick movements in the rostral supplementary motor area and right dorsolateral prefrontal cortex at 18 months post-graft but not at 6.5 months [30]. More recently, single unit recordings in a patient transplanted 10 years prior have demonstrated long-term electrophysiological changes in neurons of the globus pallidus interna (GPi). While oscillatory activity remained abnormal, compared to non-transplanted individuals in the “OFF-state”, resting discharge rates were significantly reduced and voluntary hand movements were associated with marked inhibition of neuronal firing by ~ 63% relative to baseline. No alterations were observed in the external palladium; hence, beneficial modulation of GPi outflow and subsequent thalamo-cortical activity most likely originated from increased dopaminergic effect on D_1_-type striatal GABAergic medium spiny neurons within the direct basal ganglia pathway [47].

Despite showing promise, progress in foetal cell therapy was hindered when around the turn of the century, two large NIH-funded double-blind randomised controlled designs including sham controls produced no or marginal symptomatic improvement in patients receiving transplantation [46, 48]. Putaminal dopamine synthesis increased by only 20–40% [46, 48] and although ^18^F-DOPA measures were comparable between older (> 60 years) and younger (< 60 years) patients, only in the younger group did changes in putaminal ^18^F-DOPA uptake post-surgery correlate with changes in UPDRS [48, 49]. It is worth noting that the sham-surgery groups continued to deteriorate both in terms of motor severity and ^18^F-DOPA uptake [46, 48], which opposes suggestions of placebo effects in prior open-label studies and raises further questions regarding the impact of differing trial methodology on patient outcome. Nonetheless, these data raise the possibility of a critical threshold of graft survival below which symptomatic improvement may be negligible and that the host age could to some degree modulate the extent to which or length of time taken for the implants to become functional [50].

Disease severity may also play a role in determining therapeutic effect. Two studies retrospectively analysing the extent of denervation in patients prior to transplant have shown that individuals displaying ^18^F-DOPA reductions extending to the ventral striatum were less likely to derive clinical benefit [22, 41]. In the substantia nigra, ^18^F-DOPA continues to decrease post-surgery, indicating that striatal grafts despite boosting striatal DA synthesis do not prevent the continual degeneration of native nigrostriatal neurons [41]. These findings highlight the importance of patient selection and underline the utility of ^18^F-DOPA as a potential screening tool for objectively determining disease progression. It would seem that foetal cell therapy may not be appropriate for second-line use, after patients in advanced disease stages exhibit inadequate response to dopaminergic drugs.

In addition to the inadequate clinical improvement in blinded trials, were reports of patients exhibiting abnormal involuntary movements in the “OFF-state”, emerging as early as 5 months after grafting which sometimes necessitated additional surgical intervention [21, 46, 48, 51, 52]. These movements, later termed graft-induced dyskinesia (GID) [53], were in fact noted in some patients from open label trials [54–56] but for the most part overlooked, perhaps due to the lack of sensitive and specific assessment tools for their detection in milder cases [53]. Initially, it was suggested that continued fibre outgrowth from transplantation sites could be responsible for an eventual excess of dopamine [48]. Indeed, surplus DA could cause over-inhibition of GPi outflow in a topographically unspecific manner, as revealed by single unit recordings, as well as increases in irregular discharge patterns akin to the electrophysiological properties observed for GPi neurons in non-transplanted patients during the “ON-state” [47]. However, the degree of reinnervation as assessed using ^18^F-DOPA does not correlate with GID severity [46, 51, 52] and is comparable between patients with and without GID [46, 52]. The possibility of excessive dopamine release has been addressed using ^11^C-raclopride following intravenous administration of methamphetamine. In grafted patients who had developed GIDs, Piccini et al. found a 9.55% decrease in putaminal binding potential following drug-induced DA release relative to following saline, as compared to 25.2% in healthy individuals and 6.84% in non-grafted PD patients [41, 57]. There was no association between the degree of drug-induced ^11^C-raclopride displacement and GID severity [41]. Moreover, in one case study of a patient reportedly unaffected by GID, ^11^C-raclopride binding indicated a normalisation of dopamine release (26.6% decrease compared to saline) in the transplanted putamen [29]. Taken together, these imaging data demonstrate that GID is unlikely to be associated with excessive dopamine synthesis or release from presynaptic terminals of grafted DA neurons.

The site of cell deposition and patchy or uneven reinnervation has also been suggested as possible factors for the emergence of GID [46, 48, 52, 53, 58]. Ma et al. employing parametric and sub-regional analysis of ^18^F-DOPA uptake in transplanted patients found that individuals who developed GID appeared to have significantly greater post-operative ^18^F-DOPA increases in the relatively preserved ventral putamen as compared to patients who attained good clinical benefit but did not develop GID [21], supporting earlier suggestions that putaminal grafting should be weighted towards the more denervated dorsal aspect [48]. Another ^18^F-DOPA study indicated that patients who were deemed to have the best global ordered outcome score, a composite metric pertaining to the relative significance of clinical benefit versus side effects, exhibited preservation of ventral striatal ^18^F-DOPA uptake. In contrast, those with low global ordered outcome score showed significant denervation of the ventral striatum either prior to or within the first 2 years of surgery [41]. This may indicate that the balance between clinical benefit and GID may be in part sensitive to the pre-operative ventral striatal dopaminergic state and that cell deposition strategies should entail case-by-case evaluations to promote more homogenous reinnervation.

**Fig. 1:**
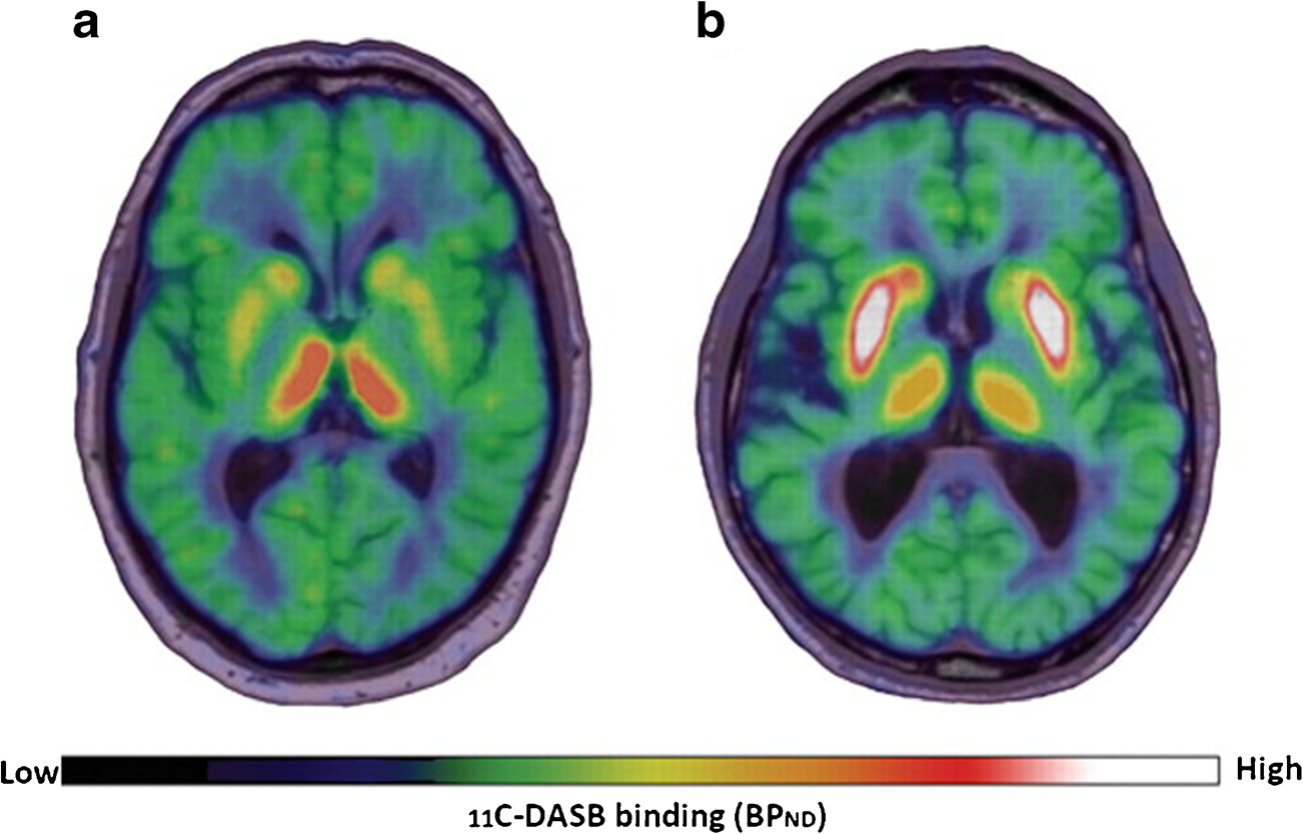
Role of striatal serotonergic hyperinnervation in graft-induced dyskinesia in Parkinson’s disease. ^11^C-DASB PET summation images of two Parkinson’s disease (PD) patients, co-registered on their individual MRI brain, showing their serotonin transporter (SERT) binding. **a** A 65-year-old male PD patient with 16 years disease duration and motor fluctuations, exhibiting reduced striatal ^11^C-DASB binding. **b** A 65-year-old male PD patient who received bilateral foetal striatal transplantation 16 years ago, experiencing graft-induced dyskinesia despite not taking any dopaminergic medications. His ^11^C-DASB PET showed significantly increased SERT binding in the striatum. Modified from Politis et al. [39]

More recently, attention has shifted towards grafted cell composition with particular focus on the subsequent growth of serotonergic neurons [27], which are known to be capable of converting l-DOPA and storing and releasing dopamine in an activity-dependent manner. Critically, the absence of necessary D_2_-autoreceptor-mediated feedback and dopamine transporters on serotonergic terminals ultimately leads to dysregulated release and excessive swings in extracellular DA levels that could potentially cause involuntary and uncontrollable movement [59–62]. Politis et al. tested this hypothesis in vivo in three transplant case studies of patients who had derived favourable long-term motor benefits with concomitant normalisation of dopamine synthesis (^18^F-DOPA) and release (^11^C-raclopride) but who also developed severe GIDs [37, 39]. Using ^11^C-DASB, which binds selectively to the serotonin transporter (SERT), a membrane protein that facilitates the reuptake of serotonin back into the presynaptic terminal, patients were found to exhibit increases in putaminal SERT expression by 46–172 and 106–285% of the mean binding values calculated for healthy controls and non-transplanted advanced PD patients respectively (Fig. 1) [37, 39]. In all three cases, administration of the 5-HT_1A_ agonist (buspirone), which dampens serotonergic neurotransmission by autoreceptor stimulation, substantially attenuated GID severity by ~ 75% while UPDRS-based motor severity measures remained unchanged. While these findings support serotonergic involvement, they do not sufficiently explain why the levels of serotonergic hyperinnervation differed between two patients (46 vs. 77%) who reportedly displayed comparable GID severity [37, 39]. In theory, dopamine transmission should not entail serotonergic involvement if the density of dopaminergic neurons in the local environment is substantial. Indeed, when considering the ratio of ^11^C-DASB binding to ^18^F-DOPA uptake, both patients exhibited similar elevations of 140 and 146% as compared to ratios in healthy individuals [37, 39].

Although data are limited and do not involve grafted individuals who did not develop GID, they appear to suggest that it is the relative density of serotonergic to dopaminergic innervation that dictates the degree of dopamine mishandling rather than serotonergic hyperinnervation as a sole causal factor. Clearly, improvements in cell sorting methodologies would be needed to reduce the risk of side effects, but so too would more comprehensive patient selection. For instance, in one open-label cohort, a near significant negative correlation was found between pre-operative putaminal ^18^F-DOPA and GID whereas no relationships were identified with post-operative ^18^F-DOPA [51]. Although this was not replicated in a blinded cohort [52], it is worth considering the exclusion of advanced patients, in whom severe dopaminergic denervation has already occurred and who may be predisposed to attaining deleteriously imbalanced post-operative 5-HT-to-DA neuronal ratios.

Contrary to this, ^11^C-DASB binding in the raphe nuclei and several extrastriatal regions receiving dense serotonergic innervation including the amygdala, thalamus, insula, cingulate, and prefrontal cortices continues to decrease following surgery [38]. Thus, while maintaining a healthy striatal 5-HT-to-DA balance may be necessary to prevent movement-related side effects, without additional targeted grafting of 5-HT neuroblasts, foetal cell therapy may not be enough to alleviate PD symptomatology where serotonin is known to play a role, such as tremor amplitude and constancy [63] and non-motor complications including depression [64–66] and fatigue [67], all of which may have a profound effect on overall quality of life.

Collectively, the imaging data obtained to date has provided in vivo evidence that foetal cell transplants can survive, produce, and release dopamine in the host brain and functionally integrate to an extent which can result in significant long-term motor improvements. Side effects such as graft-induced dyskinesias may have emerged in some cases as a consequence of patchy innervation, inappropriate targeting, impure cell suspensions, and/or the selection of advanced patients in whom pre-operative dopaminergic innervation levels were severely compromised. In vivo imaging has been and will continue to be paramount in monitoring the progress of patients receiving cell therapy and for providing an objective tool for screening patients with high likelihood of deriving clinical benefit. The use of magnetic resonance imaging has so far been limited but would offer significant insight into the behaviour of the grafted cells post-surgery and their ability to form functional connections within the effective motor network. Other PET ligands such as those that target the peripheral benzodiazepine receptor or TSPO (e.g. ^11^C-(*R*)-PK11195 and ^11^C-PBR28) found on activated microglia should be considered in future trials for evaluating the degree of immunological reactivity at and around the transplant sites which, based on histopathological and ^18^F-DOPA PET examinations, has been proposed by some as a possible hindrance to long-term clinical efficacy upon premature immunosuppressive withdrawal [41, 46, 48, 68–70]. Moreover, given reports of grafted neurons displaying Lewy pathology at post-mortem [68, 69, 71•, 72, 73••], monitoring α-synuclein and its aggregation over time is another important consideration. Despite some promising first reports on ^11^C-BF-227 [74, 75] and ^125^I-SIL23 [76], synthesis of PET radioligands with high affinity and selectivity for α-synuclein has presented as a major challenge [77•], though recent work into their development and the identification of new compounds e.g. [78] give hope that this could soon be available for use in future clinical trials.

### Neurotrophic Factors in Parkinson’s Disease

A handful of clinical trials have been performed to investigate the efficacy of neurotrophic factors in PD. Glial cell line-derived neurotrophic factor (GDNF) is a dopaminergic neurotrophic factor. Initial open-labelled pilot study of five PD patients showed both clinical and ^18^F-DOPA PET improvement following intraputaminal delivery of recombinant human GDNF [79]. However, a subsequent multi-centre double-blind randomised-controlled study showed no clinical benefit of GDNF treatment, despite an increase in striatal ^8^F-DOPA PET uptake [80]. Limited diffusion of GDNF in the putamen and placebo effect were among the factors cited for the differences in outcome between the open-labelled and double-blind studies. One patient from the initial pilot study demonstrated persistent clinical and ^18^F-DOPA PET improvement despite cessation of GDNF infusion for 3 years [81], suggesting that heterogeneity in patient responses, similar to that seen in PD transplantation studies, may also play a part in the negative outcome in the multi-centre study.

Neurturin is a naturally occurring structural and functional analogue of GDNF [82]. Randomised controlled studies injecting adeno-associated virus type 2 vector expressing neurturin into the putamen [83], and into both putamen and substantia nigra [84], of PD patients also failed to reach their primary end points. No functional imaging was undertaken in both of these studies.

### Neural Transplantation in Huntington’s Disease

The pathological hallmark of HD is progressive and severe atrophy of the striatum. Medium spiny projection neurons of the striatum, which express dopamine receptors and correspond to 90–95% of the striatal neuronal population, are preferentially lost in HD [85]. As the disease progresses, cerebral cortex, subcortical white matter, and other brainstem and basal ganglia structures are also affected.

^11^C-raclopride binding to striatal neurons of HD patients provides a marker of disease progression and correlates cross-sectionally with disability scores on the Unified Huntington’s Disease Rating Scale (UHDRS) [86]. In early-mid stage HD, the striatal D_2_ receptor binding, as measured by ^11^C-raclopride PET, fell by 5–6.3% per annum [87, 88]. PET studies on rodent models of HD which have received implantation of foetal striatal cells have shown improvement in striatal ^11^C-raclopride binding, due to the expression of dopamine D_2_ receptors by the surviving grafts. This improvement correlated with graft survival, particularly with the striatal-like tissues within the grafts (the “P zones”), and functional improvement [89]. It is the recommended functional imaging modality to assess graft survival for the Core Assessment Program for Intracerebral Transplantations in Huntington’s Disease (CAPIT-HD) assessment battery [90].

^18^F-fluorodeoxyglucose (^18^F-FDG) PET, which measures cerebral glucose metabolism, has also been used in a number of HD transplantation studies to assess graft survival. However, ^18^F-FDG uptake mainly reflects cellular metabolism and synaptic activity, which may be increased by a glial reaction to grafting and hence does not specifically indicate striatal graft survival. ^18^F-FDG does have an advantage over ^11^C-raclopride in that neuroleptics, which are frequently prescribed for HD patients for their chorea or psychosis, can interfere with D_2_ binding [91], but they do not affect cerebral glucose metabolism.

Following the initial successes of open-labelled PD transplantation studies as discussed in previous section, several HD neural transplantation studies were carried out, where foetal striatal allografts derived from either lateral ganglionic or whole ganglionic eminences were implanted into host striata. In one of the earlier UK pilot studies which included two HD subjects who received foetal striatal transplantation, one had sustained clinical improvement over 5 years which was accompanied by corresponding improvement in the striatal ^11^C-raclopride binding, indicating graft survival and functional integration. The other implanted patients did not improve and the ^11^C-raclopride continued to decline at a rate commensurate with non-implanted HD controls [92].

A French study based in Creteil showed clinical and ^18^F-FDG PET stabilisation/improvement for up to 4 years in three out of five patients transplanted with foetal striatal allografts derived from whole ganglionic eminence [93, 94]. In these three patients, clinical improvement was associated with an improvement in striatal glucose metabolic rate and a reduction in cortical hypometabolism, suggesting a partial restoration of the cortico-striatal loop function [95]. However, all of them gradually deteriorated after 4 years. The other two patients showed no clinical or PET improvement after transplantation.

In a US study, seven HD patients who received foetal striatal transplantation were assessed with ^11^C-SCH23390, ^11^C-raclopride, and ^18^F-FDG PET to measure striatal D_1_ and D_2_ binding and regional cerebral glucose metabolism respectively [96, 97]. There was no clinical or PET improvement in all seven patients, despite post-mortem evidence of graft survival with striatal tissue and extension of host-derived dopaminergic fibres into the grafts in one patient who died of cardiac disease 18 months post-transplantation [98]. There was also no evidence of graft rejection, and the grafts were not affected by HD pathology. A follow-up study involving post-mortem report of three more patients showed that there was evidence of graft survival and differentiation into medium spiny neurons in two of the patients [99]. The medium spiny neurons also received glutamatergic projection from cortical neurons. However, the graft survival was attenuated over long term and there was evidence of neuronal degeneration particularly involving medium spiny neurons. Microglial inflammatory changes were also seen in the grafts which appeared to target the neuronal components of the grafts. These findings suggest selective vulnerability of medium spiny neurons to cellular stressors, such as excitotoxicity and microglial activation, rather than the involvement of grafted neurons by primary HD pathology, as no nuclear inclusion or huntingtin protein was detected in these grafts [100].

In a German study, ten HD patients received foetal striatal transplantation [101]. Half of them developed HLA-antibodies of class I and II post-transplantation. Some of them developed the antibodies while taking immunosuppressants, while others developed after cessation of immunosuppression. Only one transplanted patient demonstrated transient clinical improvement for 24 months post-transplantation, and this was despite the presence of HLA-antibodies post-transplantation. None of the patients, however, demonstrated an increase in cerebral or striatal ^18^F-FDG PET uptake.

In a more recently published Italian study, ten HD patients received foetal striatal transplantation. Compared with non-transplanted HD controls, the transplanted patients demonstrated a slower slope of decline in their motor and cognitive assessments [102]. There was also increased striatal ^18^F-FDG PET uptake in the transplanted group up to 4 years. Three of the ten patients also received [^123^I]-iodobenzamide SPECT to image D_2_ status, and there was an increase in striatal D_2_ binding at 12 months post-operatively compared to baseline.

In another UK study, no clinical or ^11^C-raclopride PET improvement was seen in all five HD subjects implanted with foetal striatal grafts [103]. One of the transplanted subjects also received ^11^C-(*R*)-PK11195 PET pre- and post-implantation to assess microglial activation. In this subject, the level of microglial activation in the striatum was higher than a group of non-grafted HD controls 1 year post-implantation. The persistent microglial activation around the graft was detected by post-mortem in this particular patient 12 years post-transplant, indicating a possible role of microglial activation in the survival of implanted neural tissue (manuscript submitted).

Overall, the outcome of HD neural transplantation studies was heterogeneous. For the few transplanted subjects who demonstrated clinical improvement, there was generally a corresponding improvement in their PET markers of graft survival or activity, apart from one subject in the German study who showed transient clinical improvement [101]. Conversely, none of the patients who did not improve clinically displayed an improvement in their PET parameters. This suggests that despite post-mortem evidence of graft survival in most of the patients examined (incidentally, none of those who underwent post-mortem examination experienced improvement after transplantation), insufficient graft survival may partially explain the lack of PET and clinical improvement. There are a number of factors that are thought to affect graft survival, including microglial activation, alloimmunisation, lack of graft vascularisation, and loss of trophic support due to atrophic astrocytes around the graft [104, 105•]. However, as PD transplantation studies tell us, evidence of significant graft survival might not necessarily lead to clinical improvement. It may require a more complete integration of graft into the host circuitry and normalisation of neurotransmitters release in the host environment.

## Conclusion

Imaging and pathological findings have informed us that graft survival does not necessarily translate to clinical improvement in neural transplantation studies. The heterogeneity in patient responses may partially contribute to the differences in outcome between open-labelled and subsequent double-blind studies in PD. PET findings can help to identify and select patients who are more likely to do well in neural transplantation studies. They also highlight the importance of restoring normal balance of neurotransmitters in the host brain. Clinical improvement in HD transplantation studies is generally less impressive than that seen in PD open-labelled studies. Variable graft survival and host inflammatory response, as revealed by PET, could play an important part.

The lessons learned from imaging findings in previous neural transplantation studies are being used to refine protocol and patient selection for future studies (e.g. TRANSEURO for PD [103] and MIG-HD for HD [106]). The shift in interest from foetal progenitor cells to stem cells has also rejuvenated the neural transplantation field [107]. Functional imaging will continue to play an important role in assessing the outcome of these trials.
